# Evaluation of the antioxidant and longevity-promoting effects of white tea extract in *Drosophila melanogaster*

**DOI:** 10.3389/fnut.2025.1702854

**Published:** 2025-11-14

**Authors:** Yuhua Yang, Tingting Ye, Jingyi Yu, Li Fan, Chunhua Ma, Bo Zhang, Thuan-Chew Tan

**Affiliations:** 1 College of Tea and Food Science/Fujian Key Laboratory of Big Data Application and Intellectualization for Tea Industry, Wuyi University, Wuyishan, China; 2 Food Technology Division, School of Industrial Technology, Universiti Sains Malaysia, Penang, Malaysia; 3 The People’s Hospital Affiliated to Fujian University of Traditional Chinese Medicine, Fuzhou, China; 4 Renewable Biomass Transformation Cluster, School of Industrial Technology, Universiti Sains Malaysia, Penang, Malaysia

**Keywords:** white tea extract, *Drosophila melanogaster*, longevity extension, oxidative stress-related, gene expression

## Abstract

**Introduction:**

White tea is known for its health benefits, especially its strong antioxidant activity.

**Methods:**

Based on previous studies showing white tea extract (WTE) has antioxidant effects *in vitro*, this research further evaluated its potential to delay aging in *Drosophila melanogaster*.

**Results:**

The results showed that dietary supplementation with WTE significantly (*p* < 0.05) increased both the mean lifespan (T_m_) and longest survival time (T_max_) of *Drosophila melanogaster*. T_m_ levels rose by 15.41% in males and 30.39% in females at 3 mg/mL concentration, while the T_max_ showed increases of 21.05 and 32.27%, respectively. WTE also improved the flies’ climbing ability and their resistance to hydrogen peroxide-induced stress. It markedly (*p* < 0.05) decreased malondialdehyde (MDA) concentrations while enhancing the activities of antioxidant enzymes, such as total superoxide dismutase (T-SOD) and catalase (CAT), in a dose-responsive manner. Gene expression analysis indicated that WTE significantly upregulated the antioxidant-related genes SOD1, SOD2, and CAT, while suppressing the expression of the aging-related MTH gene.

**Discussion:**

Overall, WTE helps delay aging in fruit flies by boosting antioxidant defenses and reducing oxidative damage. These results suggest its potential use as a natural antioxidant and anti-aging ingredient in food and health products.

## 1 Introduction

Aging is a complex and inevitable biological process marked by a gradual decline in physiological functions across molecular, cellular, tissue, and organ levels (1, 2). Key features include reduced cellular activity, weakened immunity, metabolic slowdown, structural deterioration of organs, and genetic damage (3). These changes impair mobility, reproduction, and lifespan, largely driven by free radicals—reactive by-products of oxidative stress that contribute to aging and disease development (4, 5). The antioxidant defense mechanism of the body involves essential enzymes such as superoxide dismutase (SOD), catalase (CAT), glutathione peroxidase (GPx), and glutathione reductase (GR), along with dietary antioxidants like vitamin C, polyphenols, flavonoids, and carotenoids, plays a vital role in neutralizing free radicals and maintaining redox balance (6). However, excessive ROS production can overwhelm this system, resulting in cellular damage and accelerated aging (7–9). Given the role of oxidative stress in aging, strategies to reduce ROS and enhance antioxidant defenses are of growing interest. Natural antioxidant-rich foods are increasingly valued for their potential to delay aging. Among them, white tea flavonoids have demonstrated promising antioxidant properties and may offer protective effects against oxidative stress. White tea contains a variety of bioactive components that are advantageous to human health, such as flavonoids, amino acids, polyphenols, polysaccharides, and vitamins. Previous research has shown that flavonoids exert diverse pharmacological effects by targeting multiple biological pathways, contributing to anti-inflammatory, antioxidant, glycemic-regulating, anti-fatigue, and antidepressant activities (10–13). As a primary bioactive component among tea polyphenols—making up approximately one-quarter of tea’s dry weight—flavonoids play a critical role in tea’s health-promoting properties (14). Consequently, beyond their traditional use in beverages, tea leaves hold strong potential as plant-derived functional ingredients for delaying aging. Despite this, the longevity-enhancing effects and underlying mechanisms of white tea flavonoids remain insufficiently explored. This suggests that white tea flavonoid compounds may serve as promising candidates for mitigating senescence and combating age-related disorders.

Fruit flies (*Drosophila melanogaster*) are a distinct invertebrate model that exhibits a functional heart. Notably, their cardiac performance, akin to that of humans, diminishes as they age (15). Genetically, approximately 70% of genes associated with human diseases possess homologs in *D. melanogaster*, rendering it an effective model for investigating aging and age-related diseases at the genetic level (16, 17). Due to its short lifespan, well-characterized genome, and conserved signaling pathways, *D. melanogaster* has been extensively used to evaluate the impact of dietary interventions on longevity. Previous studies have demonstrated that certain plant extracts can effectively promote lifespan in *D. melanogaster*. For example, supplementation with Aronia berry extract was shown to enhance locomotor function and extend lifespan by increasing antioxidant enzyme activities and upregulating stress response genes (18). Similarly, purple nutsedge (*Cyperus rotundus*) extract was reported to prolong lifespan through reducing oxidative damage, enhancing stress resistance, and mitigating heavy metal toxicity (19). These findings highlight the suitability of *D. melanogaster* as a model organism for assessing the anti-aging potential of natural compounds.

Dietary interventions are widely recognized as key modulators of aging and longevity. Various nutritional strategies, including specific dietary components and time-restricted feeding, have been shown to significantly influence lifespan across species (20–22). In this context, *D. melanogaster* has emerged as a widely accepted model for studying dietary effects on aging and related physiological processes. For instance, polysaccharides derived from poplar mushroom (*Agrocybe aegerita*) exert anti-aging effects in fruit flies by modulating oxidative imbalance and intestinal microbiota (23). Similarly, Astragalus water extract has been reported to enhance degradation functions and extend lifespan through antioxidant pathways, protecting against oxidative agents such as hydrogen peroxide and paraquat (24, 25). Despite growing interest, *in vivo* studies on natural antioxidants and their anti-aging mechanisms remain limited. To our knowledge, white tea, abundant in flavonoids with potent antioxidant properties, is postulated to contribute to longevity. However, its in vivo efficacy has not yet been comprehensively elucidated. Therefore, the present study aims to investigate the antioxidant activity and anti-aging effects of white tea extract (WTE) in *D. melanogaster*. This research provides foundational insight into the mechanisms by which WTE may mitigate oxidative stress and delay aging *in vivo*.

## 2 Materials and methods

### 2.1 Sample solutions preparation

White tea was procured from Fujian Province Jiulong White Tea Co., Ltd. (Fujian, China), identified as the *Camellia sinensis* cultivar ‘Shui Xian’ cultivated in Zhenghe County, Fujian Province. The white tea extract (WTE) was obtained through an optimized procedure combining ultrasound-assisted extraction of flavonoids and purification with macroporous resin (60). In brief, ultrasonic extraction was carried out by dispersing 1 g of tea powder in 60% ethanol (v/v) at a solid-to-liquid ratio of 1:42 (g/mL), using an ultrasonic water bath (Shumei Ultrasonic Instrument, KQ3200DE, Kunshan, China) operating at 180 W for 1 h. Following extraction, the mixture was centrifuged at 2,795 × g for 10 min (Zhongke Zhongjia Instrument, HC-2514, Anhui, China). The supernatant was concentrated under reduced pressure with a rotary evaporating apparatus (Zhuocheng Instrument, RE-201D, Shanghai, China) set at 60 °C and 15 rpm, then freeze-dried (Buchi Instrument, L-200, Switzerland, Switzerland) to yield a unrefined WTE powder.

The unrefined extract was dissolved in distilled water and then purified using a column packed with AB-8 macroporous resin (diameter: 1.6 cm; length: 30 cm; resin bed height: 20 cm) at 25 °C, operated at a flow rate of 1.5 bed volumes per minute. Elution was performed with 80% ethanol, and the eluate was subsequently concentrated under vacuum via rotary evaporation at 60 °C and 150 rpm. The condensed eluate was subjected to freeze-dried to yield the purified white tea extract (WTE) in powder form.

### 2.2 Analysis of flavonoid constituents in WTE

The flavonoid profile of white tea extract (WTE) was analyzed by high-performance liquid chromatography (UPLC) using an e2695 Waters system (Singapore Waters Instrument, Alliance e2695, Singapore). For the analysis, 0.2 mg of WTE was solubilized in 1 mL of methanolic solution (27). Reference standards—such as rutin, myricetin, quercetin, and kaempferol—were used for comparison—were accurately weighed (totaling 0.2 mg), placed into a 1 mL microcentrifuge tube, and solubilized in 1 mL of wood alcohol. Both the samples and standards were isolated using a DIKMA Diamonsil Plus C18 column (4.6 × 250 mm, 5 μm particle size) kept at 30 °C. The liquid phase comprised solvent A (acetonitrile) and solvent B (0.05% aqueous phosphoric acid solution, pH 3), with gradient elution programmed as follows: from 0 to 20 min, 12 to 30% solvent A; 20 to 32 min, held at 30% A; 32 to 36 min, increased from 30 to 80% A; 36 to 37 min, decreased from 80% back to 12% A; and 37 to 45 min, maintained at 12% A. The flow rate was set at 1.0 mL/min, and detection was performed at 260 nm.

### 2.3 Evaluation of antioxidant activity *in vitro*

#### 2.3.1 Hydroxyl radical scavenging activity evaluation

The ability of the extracts and vitamin C (as a positive control) to scavenge hydroxyl radicals was appraised following the procedure outlined by Zabik et al. (28). Samples at concentrations of 0.1, 0.2, 0.4, 0.6, 0.8, and 1.0 mg/mL (1.0 mL each) were mixed with 1 mL of 7.5 mM ferrous sulfate (FeSO₄) reagent, 2 mL of 5 mM PBS (phosphate-buffered saline) (pH 7.4), and 1 mL of 0.1% hydrogen peroxide (H₂O₂). The reaction preparation was maintained at 37 °C for 1 h. After incubation, the preparations were cooled under a stream of running water, and purified water was supplemented to bring the final liquid volume to 10 mL. The absorption value of the upper phase was noted at 510 nm using a UV–visible absorbance spectrometer (Yuanxi Instrument, UV-6100, Shanghai, China). Control measurements included a blank solution, where purified water replaced the test solution, and a control sample, where purified water was used instead of H₂O₂. Both followed the same procedural steps. The hydroxyl radical scavenging capacity was calculated using the Equation 1:

$$\begin{array}{l} \mathrm{Hydroxyl radical scavenging capacity}\;\left(\%\right)\\ =\left[\left(A_{B}–A_{S}\right)/\left(A_{C}–A_{S}\right)\right]\times 100 \end{array}$$ (1)

where, *A*_S_, *A*_C_, and *A*_B_ denote the absorbance values of the sample solution, the reference solution, and the blank at 510 nm, respectively.

#### 2.3.2 Superoxide radical neutralization evaluation

The superoxide anion radical scavenging activity of the extracts was evaluated using the pyrogallol autoxidation method (29). After preheating for 20 min, 4.5 mL of 50 mM tris–HCl buffer (pH 8.2) was mixed with 1.0 mL of sample solutions at 0.1, 0.2, 0.4, 0.6, 0.8, and 1.0 mg/mL content and 0.4 mL of 25 mM pyrogallol solution. The reaction was carried out at 25 °C for 5 min, after which 1 mL of 8 mM HCl was added to terminate the reaction. The absorbance of the resulting supernatants was measured at 325 nm using the UV-6100 UV–visible spectrophotometer. Distilled water was used as a blank control in place of the sample. The superoxide radical scavenging capacity was calculated using Equation 2.

$$\begin{array}{l} \mathrm{Superoxide radical scavenging capacity}\;\left(\%\right)\\ =\left[\left(A_{B}–A_{S}\right)/A_{B}\right]\times 100 \end{array}$$ (2)

where, *A*_S_ and *A*_B_ denote the absorbance values of the sample solution, the reference solution, and the blank at 325 nm, respectively.

#### 2.3.3 DPPH antioxidant activity evaluation

WTE was prepared in purified water to obtain sample solutions at various concentrations. The DPPH radical scavenging activity was assessed following a modified protocol based on Abdelfattah et al. (30). Specifically, three reaction mixtures were prepared: Sample solutions were prepared by mixing 2 mL of the sample at 0.1, 0.2, 0.4, 0.6, 0.8, and 1.0 mg/mL with 2 mL of 0.3 mM DPPH solution in 95% ethanol. The reference solution consisted of 2 mL of ethanol combined with 2 mL of DPPH, while the blank comprised 2 mL of the sample mixed with 2 mL of ethanol. All mixtures were maintained in a dark place (25 °C) for 30 min. Absorbance data were noted at 517 nm using the UV-6100 UV–visible spectrophotometer. The DPPH scavenging capacity was calculated using Equation 3.

$$\mathrm{DPPH scavenging capacity}\;\left(\%\right)=\left[1–\left(A_{s}–A_{R}\right)/A_{B}\right]\times 100$$ (3)

where, *A*_S_, *A*_R_, and *A*_B_ denote the absorbance values of the sample solution, the reference solution, and the blank at 517 nm, respectively.

#### 2.3.4 ABTS antioxidant activity evaluation

ABTS radical scavenging activity was measured with slight modifications based on the method described by Dan et al. (24). Sample solutions were prepared at concentrations of 10, 20, 40, 60, 80, and 100 μg/mL. Equal volumes of 7 mM ABTS solution and 2.45 mM potassium persulfate (K₂S₂O₈) were mixed and allowed to react in the dark for 16 h. The resulting ABTS•^+^ solution was diluted with 5 mM phosphate buffer (pH 7.4) until an absorbance of 0.70 ± 0.02 at 734 nm was reached. Then, 0.4 mL of sample solution was mixed with 4 mL of the prepared ABTS solution and incubated in the dark for 5 min. Absorbance was recorded at 734 nm using the UV-6100 UV–visible spectrophotometer. Distilled water was used instead of the sample in the blank control. The ABTS radical scavenging capacity of the samples was calculated using Equation 4:

$$\mathrm{ABTS scavenging capacity}\;\left(\%\right)=\left(1–A_{S}/A_{B}\right)\times 100$$ (4)

where, *A*_S_ and *A*_B_ denote the absorbance values of the sample solution and the blank at 734 nm, respectively.

### 2.4 *Drosophila* strain and feeding regimen

Wild-type fruit flies (*D. melanogaster* w^1118^ strain) were obtained from the Institute of Marine Science (Xiamen, Fujian, China). The fruit flies were maintained in an incubator set at 25 ± 1 °C with 60% humidity and a 12-h light/dark cycle. The standard culture medium was prepared in glass containers and composed of a basal diet containing 27 g ground corn, 13 g cane sugar, 1.5 g agar, 1.5 g yeast, and 150 mL distilled water, with the addition of 1.2 mL propanoic acid to prohibit mold development (31).

### 2.5 Longevity evaluation

Fruit flies were collected within 8 h post-eclosion and randomly assigned to four groups for each sex, with a total of 100 males and 100 females (total flies: 800, 20 flies per tube) (1). The control group (CTL) was maintained on the standard diet, while treatment groups received the standard medium supplemented with WTE was administered at three different concentrations: Doses were set at 0.5 mg/mL for LDG, 1.0 mg/mL for MDG, and 3.0 mg/mL for HDG. Fruit flies were transferred to fresh food vials every 3 days. Mortality was recorded daily until all fruit flies had died (32, 33). Key lifespan parameters, including mean lifespan (T_m_; calculated as the mean lifespan across all groups), longest survival time (T_max_; defined as the average survival time of flies during the last 24 days), and median survival time (LT_50_; the time at which 50% of the population had died) were determined for each group. Survival curves were generated using GraphPad Prism 8.0 software, and statistical significance was assessed via the log-rank (Mantel-Cox) test.

### 2.6 Locomotor activity test

Aging is commonly associated with a decline in locomotor activity in both animals and humans. Due to their natural locomotor behavior in confined spaces, fruit flies serve as a suitable model for evaluating physical mobility through climbing ability tests. The assay was performed with modifications based on the method by Zhou et al. (34). Briefly, newly eclosed fruit flies (400 males and 400 females, with 20 individuals per tube with flies of the same sex) were reared on their respective diets. Groups of fruit flies were transferred into empty tubes and gently tapped to the bottom. For each tube, the count of fruit flies that climbed at least 7 cm within 10 s was recorded. The climbing ability was calculated according to Equation 5. This test was repeated three times on days 15, 30, and 45 of the feeding regimens, with at least a 1-min interval between trials.

$$\begin{array}{l} \mathrm{Locomotor ability}\;\left(\%\right)\\ =\left(\begin{array}{l} \mathrm{the number of flies that climbed}\;a\;\mathrm{given}\\ \mathrm{distance within}\;a\;\mathrm{set}\;\mathrm{time}\geq 7\mathrm{cm} \end{array}\right)/20\times 100 \end{array}$$ (5)

### 2.7 H₂O₂-induced stress challenge

Hydrogen peroxide (H₂O₂) is a reactive oxidant that can produce highly reactive hydroxyl radicals. It possesses dual properties, acting as both an oxidizing and reducing agent. The oxidative stress challenge was conducted following the protocol depicted by Yang et al. (26). Eclosed fruit flies (400 males and 400 females, 25 flies per tube, segregated by sex) were maintained on control or WTE-supplemented diets for 25 days. Afterwards, the flies were food-restricted for 2 h before being transferred to tubes containing engulfed filter paper soaked with 6% glucose solution and 1 mL of 30% H₂O₂. Care was taken to ensure the filter paper was thoroughly moist but without any dripping liquid. Mortality was marked every 4 h until all fruit flies had expired. The Survival rate was calculated using the Equation 6.

$$\mathrm{Survival rate}\;\left(\%\right)=\left[1-\left(\begin{array}{l} \mathrm{number of flies that}\\ \mathrm{died every}\;4\;\mathrm{hours} \end{array}\right)\right]/25\times 100$$ (6)

### 2.8 Feeding behavior evaluation

Dietary restriction has been demonstrated to affect lifespan extension. To eliminate the potential that the prolonged lifespan identified in the survival assay resulted from decreased food consumption, a gustatory assay was performed following the method of Wongchum et al. (19). Newly eclosed fruit flies (240 males and 240 females, 20 flies per tube, separated by sex) were distributed to all the groups and maintained for 6 days. The fruit flies were subsequently starved for 24 h in vials lined with Kimwipes soaked in distilled water. Following this, sodium sulfadiazine B (acid red) was incorporated into the culture medium at 0.2%, and the fruit flies were permitted to feed for 2 h. Following feeding, the fruit flies were anesthetized using anhydrous diethyl ether, and then the abdominal redness of the fruit flies was observed under a stereomicroscope. A score of 0 (no red abdomen) to 5 (fully red abdomen) abdominal redness scoring system was employed to quantify feeding, enabling comparison of food consumption among groups.

### 2.9 Measurement of antioxidant enzyme activity

Briefly, newly eclosed fruit flies (2,400 males and 2,400 females, 20 flies per tube, grouped by sex) were fed their respective diets for 30 and 45 days. After a 2-h fasting period, fruit flies were immobilized by liquid nitrogen and weighed and then preserved at −80 °C (35). For biochemical assays, a 10% tissue homogenate was prepared by homogenizing fruit flies in ice-cold physiological saline (1:49 w/v), followed by centrifugation at 2,795 × g for 15 min at 4 °C. Following the manufacturer’s protocol (Nanjing Jiancheng Bioengineering Institute, Nanjing, China), the supernatants were collected and diluted for subsequent determination of superoxide dismutase (SOD), catalase (CAT), malondialdehyde (MDA), and protein content (via Coomassie Brilliant Blue assay).

### 2.10 Evaluation of gene expression levels

Newly eclosed fruit flies (240 males and 240 females, 20 flies per tube, grouped by sex) were reared on their respective diets for 25 days (18). Samples were preserved at −80 °C using the TRIzol method. Complementary DNA (cDNA) was synthesized from isolated total RNA using the High-Capacity cDNA Reverse Transcription Kit. Quantitative qRT-PCR analysis was then conducted employing SYBR Green chemistry along with sequence-specific primers. Using Rp49 as the internal control, relative expression levels of target genes were quantified via the 2^^−ΔΔCT^ method (36). TRIzol Reagent, along with the High-Capacity cDNA Reverse Transcription Kit and SYBR Green Kit, was supplied by Sangon Biotech Co., Ltd. (Shanghai, China). Primers designed for antioxidant-related genes (as shown in Table 1) were synthesized by Sangon Biotech Co., Ltd.

**Table 1:** Real-time PCR primers sequence of genes in fruit flies (*Drosophila melanogaster*).

Gene name	Sequence (5′ to 3′)	Annealing temperature (°C)
SOD1	GCGGCGTTATTGGCATTG	53
	ACTAACAGACCACAGGCTATG	
SOD2	AGAACCTCTCGCCCAACAAG	55
	CGTGGTCAGCTCCTTTTTGAAC	
CAT	GAATGTGACGGACAACCAGGATG	53
	CGGACAGCAGGAGGACAAGG	
MTH	GAGGAGGTAAACAACAGTGAGGAAG	55
	CCACGGTAATACGACTTGCCATAG	
Rp49	CTTCATCCGCCACCAGTC	55
	GCACCAGGAACTTCTTGAATC	

### 2.11 Analysis of statistics

Unless otherwise specified, data are shown as mean ± standard deviation in triplicate (n = 3), except for the longevity analysis, locomotor activity assay, and hydrogen peroxide challenge (n = 5). Statistical analyses were performed using SPSS Statistics version 23 (IBM, Chicago, United States). Group survival curves were evaluated using Kaplan–Meier analysis, with statistical comparisons made via the log-rank test. One-factor ANOVA followed by Tukey’s *post hoc* test was applied to compare differences between group means. Statistical significance was set at *p* < 0.05, and graphs were created using GraphPad Prism 8.0 (GraphPad Software, San Diego, United States).

## 3 Results and discussion

### 3.1 Flavonoid content of WTE

**Figure 1:**
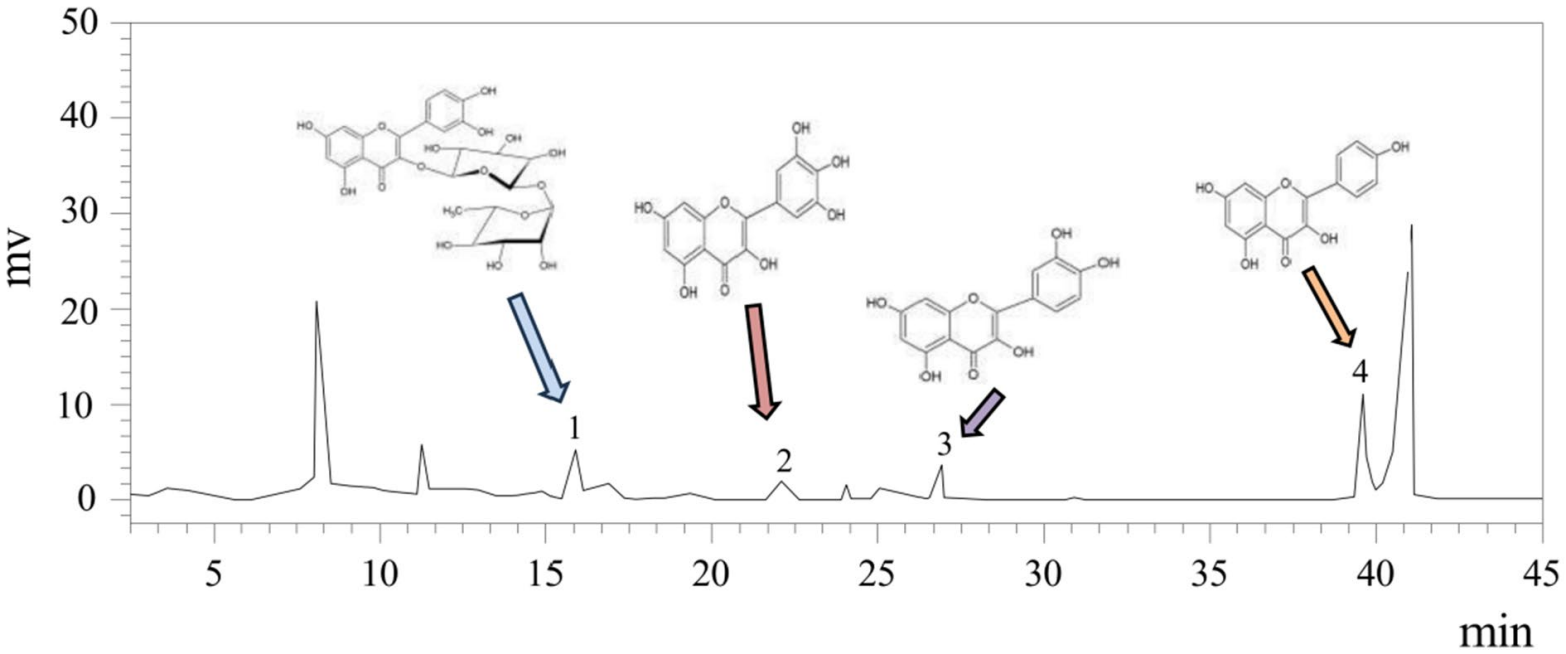
HPLC analysis of WTE identified rutin, myricetin, quercetin, and kaempferol as peaks 1–4, with other peaks remaining unidentified.

Flavonoids such as rutin, myricetin, quercetin, and kaempferol, known for their notable biological properties (37), were quantified in white tea extract (WTE) (Figure 1) using an external standard method. Quercetin and kaempferol concentrations were determined to be 1.7828 ± 0.22 μg/mg and 5.726 ± 0.31 μg/mg, respectively (Table 2). The presence of these bioactive flavonoids provides a basis for their further isolation and structural characterization. Additionally, this study supports previous reports that white tea primarily contains the flavonoids rutin, quercetin, myricetin, and kaempferol (38).

**Table 2:** The flavonoid content in the white tea extract (WTE).

Compounds	Retention time (min)	Content (μg/mg)
Rutin	15.887	2.5389 ± 0.19
Myricetin	22.356	0.9425 ± 0.10
Quercetin	26.896	1.7828 ± 0.22
Kaempferol	39.593	5.7926 ± 0.31

Data are represented as means ± standard deviations (*n*=3).

### 3.2 Evaluation of WTE’S antioxidant activity *in vitro*

**Figure 2:**
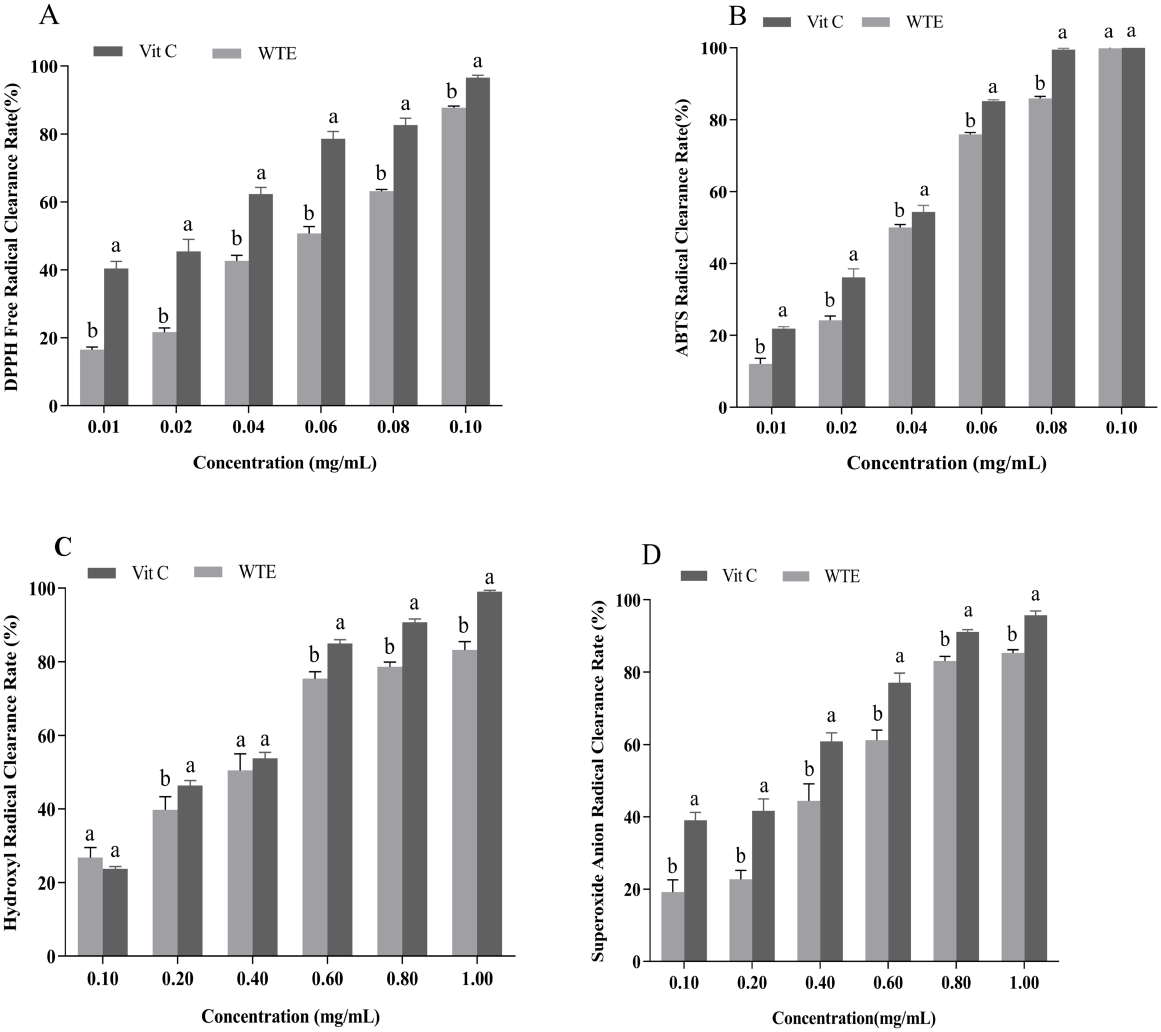
*In vitro* antioxidant activities of white tea extract (WTE), assessed by **(A)** DPPH, **(B)** ABTS, **(C)** hydroxyl, and **(D)** superoxide anion radical scavenging assays. Data are expressed as mean ± SD (n = 3). Different lowercase letters indicate significant differences between groups (*p* < 0.05). Vitamin C (Vit C) was used as the positive control.

The ability to scavenge DPPH and ABTS free radicals is commonly used as a standard approach to assess antioxidant capacity. Hydroxyl and superoxide anion radicals are classified as reactive oxygen species (ROS), can cause cellular damage, and their neutralization contributes to antioxidant effects (14, 39). In this study, the antioxidant potential of WTE was assessed by measuring its scavenging activities against DPPH (Figure 2A), ABTS radicals (Figure 2B), hydroxyl radicals (Figure 2C), and superoxide anion radicals (Figure 2D).

IC₅₀ is an important parameter for evaluating the inhibitory potency of a substance; the lower the concentration, the stronger the inhibitory effect (40). WTE exhibited IC₅₀ values of 0.02835 mg/mL and 0.04011 mg/mL for scavenging hydroxyl radicals and superoxide anion radicals, respectively. In comparison, vitamin C exhibited IC₅₀ values of 0.02472 mg/mL and 0.02051 mg/mL for the same radicals. These results highlight WTE’s strong hydroxyl radical scavenging ability. Additionally, WTE showed a clear dose-dependent scavenging effect on DPPH and ABTS radicals. Vitamin C exhibited IC₅₀ values of 0.01900 mg/mL for DPPH radicals and 0.02688 mg/mL for ABTS radicals, while WTE demonstrated IC₅₀ values of 0.04786 mg/mL and 0.03560 mg/mL for these radicals. Remarkably, at a concentration of 0.1 mg/mL, WTE achieved 100% scavenging of ABTS radicals. The data demonstrate that WTE has powerful ABTS radical neutralizing effects and considerable antioxidant potential *in vitro*, supporting its promising application as a natural antioxidant and laying the groundwork for further investigation into its senescence-inhibiting properties. In a similar study, Atak et al. (41) reported the *in vitro* antioxidant properties of three tea varieties. These varieties exhibited remarkable scavenging abilities for DPPH free radicals, with IC₅₀ values of 0.014 mg/mL for white tea, 0.035 mg/mL for green tea, and 0.016 mg/mL for handcrafted green tea. Furthermore, the antioxidant properties of green tea and turmeric extracts were evaluated in a rat model by assessing the activities of SOD and MDA levels. The results indicated that green tea exhibited an IC₅₀ value of 0.75 ± 0.16 μg/mL, while turmeric extract showed an IC₅₀ of 5.3 ± 0.4 μg/mL for reducing 50% of DPPH radicals (42).

### 3.3 WTE supplementation led to lifespan extension in fruit flies

**Figure 3:**
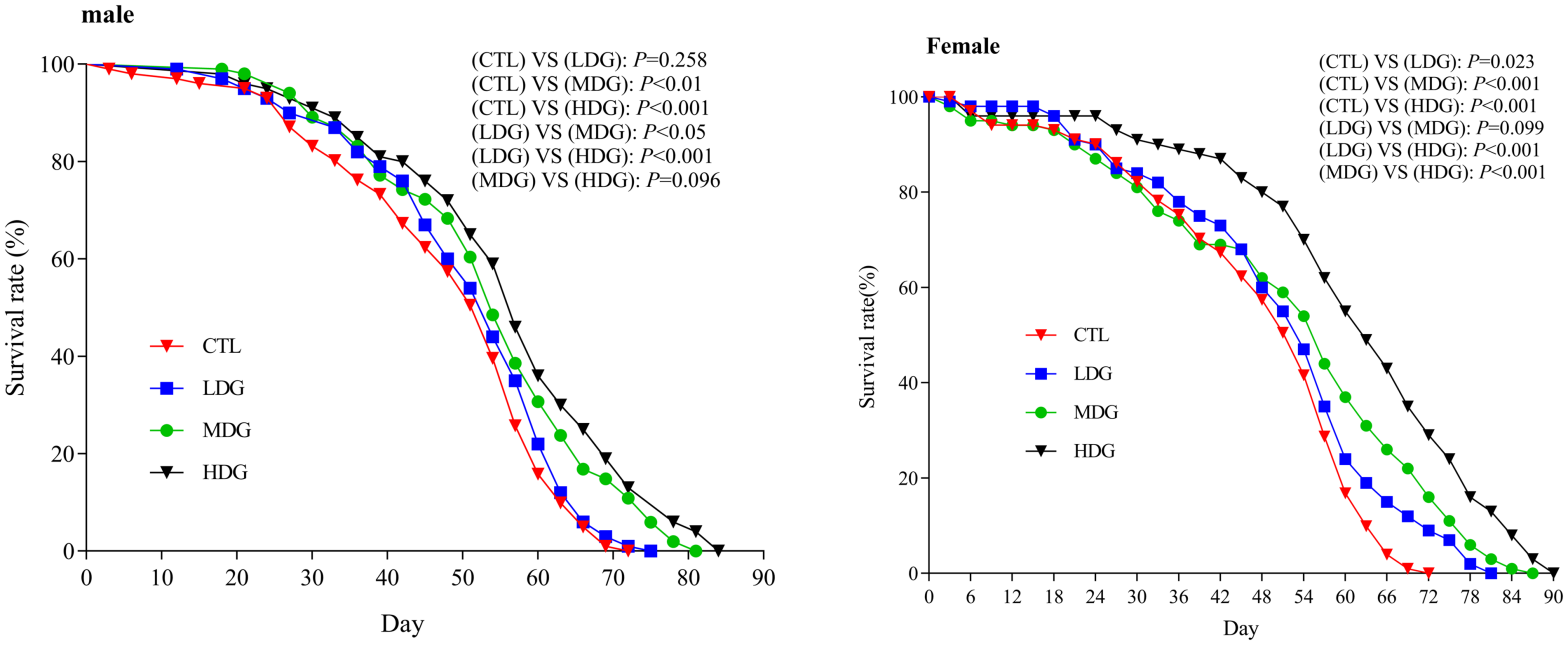
Kaplan–Meier survival curves of male and female *Drosophila melanogaster* fed diets supplemented with white tea extract (WTE) at 0.5 (LDG), 1.0 (MDG), and 3.0 mg/mL (HDG). In females, all treatment groups showed significantly extended lifespan compared to control (CTL) (LDG: *p* = 0.023; MDG: *p* < 0.001; HDG: *p* < 0.001). In males, lifespan extension was significant in MDG (*p* < 0.01) and HDG (*p* < 0.001), but not in LDG (*p* = 0.258). Data are presented as mean ± SD (*n* = 5). Survival differences were analyzed using the log-rank test.

In light of WTE’s proven antioxidant effects observed in vitro, its anti-aging effects were further validated through lifespan assays in fruit flies (Figure 3; Table 2). Relative to the control group (CTL), both the medium dose group (MDG) and high dose group (HDG) significantly (*p*<0.01) prolonged lifespan in male and female fruit flies. In males, the mean lifespan (T_m_) increased by 10.54 and 115.41%, the median survival time (LT_50_) rose by 10.66 and 15.75%, and the maximum lifespan (T_max_) prolonged by 14.91 and 21.05% for MDG and HDG, respectively (*p*<0.01). Female flies exhibited even more pronounced lifespan extensions, with T_m_ increased by 10.23 and 30.39%, LT_50_ by 8.16 and 34.48%, and T_max_ by 21.82 and 32.27% in MDG and HDG, respectively (*p*<0.01). These findings demonstrated that dietary supplementation with WTE significantly enhances longevity in fruit flies, consistent with previous reports by Han et al. (43).

This study found that female fruit flies exhibited greater longevity than males when treated with WTE, indicating that females may better tolerate higher doses of WTE (HDG), as reflected by T_max_ data (Figure 3). Furthermore, supplementation with 3.0 mg/mL WTE increased survival rates by 14.81% in males and 20% in females. WTE administration significantly enhanced LT_50_, T_m_, and T_max_ in the HDG groups (Figure 3; Table 3). These results align with the study by Yang et al. (26), which revealed that Chinese mugwort (*Artemisia argyi*) extract prolongs lifespan and enhances motor function in fruit flies. Similarly, Kang et al. (44) reported that puerarin intake contributes to survival time prolongation and enhances physical activity in male fruit flies.

**Table 3:** Effect of various doses of white tea extract (WTE) on the lifespan of male and female fruit flies (*Drosophila melanogaster*).

Sex and dosage group		50% Survival days (LT_50_, days)	Mean lifespan (T_m_, days)	Maximum lifespan (T_max_, days)
Male	CTL	47.37 ± 3.48^c^	48.66 ± 1.63^c^	68.40 ± 1.12^c^
	LDG	49.49 ± 2.33^bc^	50.79 ± 1.10^bc^	70.80 ± 1.52^c^
	MDG	52.42 ± 2.24^ab^	53.79 ± 1.02^ab^	78.60 ± 1.12^b^
	HDG	54.83 ± 2.04^a^	56.16 ± 0.97^a^	82.80 ± 1.20^a^
Female	CTL	43.36 ± 4.70^b^	47.78 ± 3.78^b^	66.00 ± 1.25^c^
	LDG	46.44 ± 2.22^b^	50.99 ± 1.50^b^	77.10 ± 1.54^b^
	MDG	46.90 ± 2.19^b^	52.67 ± 1.46^b^	80.40 ± 1.47^b^
	HDG	58.31 ± 2.37^a^	62.30 ± 1.92^a^	87.30 ± 0.87^a^

Data are represented as means ± standard deviations (*n*=5). Different lowercase letters indicate significant differences within the same group (*p*<0.05). CTL, control group; LDG, MDG, and HDG represent diets containing 0.5, 1.0, and 3.0 mg/mL of WTE, respectively.

Female fruit flies typically exhibit a longer lifespan compared to males, which can be attributed to genetic factors. Genetically, females exhibit elevated expression levels of genes associated with oxidative stress resistance, DNA repair, and longevity regulation, including superoxide dismutase (SOD), catalase (CAT), and heat shock protein 70 (HSP70). These genes enhance their cellular protection against reactive oxygen species, contributing to their extended lifespan (31, 45). Furthermore, sex-specific variations in the insulin/IGF-1 signaling (IIS) and target of rapamycin (TOR) pathways are of paramount importance. Female fruit flies generally exhibit lower IIS/TOR activity, resulting in enhanced stress resilience and an extended lifespan (46, 47). Sex-determination genes, such as *doublesex* (dsx), *transformer* (tra), and *Sex-lethal* (sxl), further influence metabolic and immune-related processes that shape the aging trajectories between the sexes (48).

### 3.4 Role of WTE supplementation on fruit fly resistance to hydrogen peroxide stress

**Figure 4:**
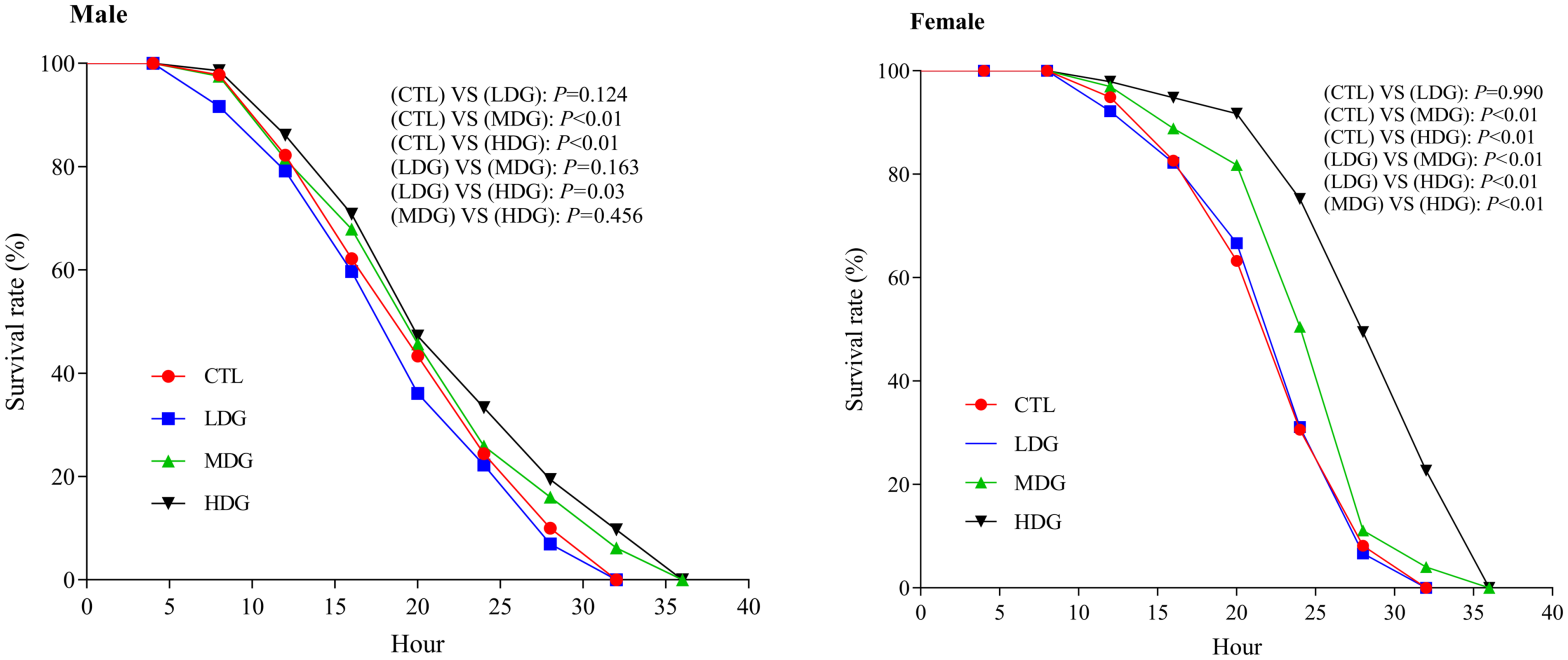
Effect of hydrogen peroxide–induced oxidative stress on the survival of *Drosophila melanogaster* fed diets containing white tea extract (WTE) at 0.5 (LDG), 1.0 (MDG), and 3.0 mg/mL (HDG). In females, MDG and HDG significantly improved survival compared to the control (CTL) (*p* < 0.01), while LDG showed no significant effect (*p* = 0.124). In males, significant differences were observed in MDG and HDG (*p* < 0.01), but not in LDG (*p* = 0.990). Data are presented as mean ± SD (*n* = 5). Survival was analyzed using the Kaplan–Meier log-rank test.

Hydrogen peroxide (H₂O₂), a potent oxidizing agent that generates hydroxyl radicals and rapidly elevates ROS levels in fruit flies, is widely used to induce oxidative stress in antioxidant studies (49). This research examined the potential of WTE to promote resistance to oxidative stress and prolong lifespan. Under H₂O₂-induced oxidative conditions, male fruit flies in the MDG and HDG groups exhibited markedly increased survival rates compared to the control group, with improvements of 5.01 and 8.24%, respectively (Figure 4). Consistent with these findings, Yang et al. (50) demonstrated that supplementation with wolf berry polysaccharides significantly lowered mortality caused by H₂O₂. These results suggest that WTE mitigates oxidative damage induced by H₂O₂ and may delay the aging process.

Notably, female fruit flies exhibited the most pronounced increase in survival under H₂O₂ stress in the MDG and HDG, with survival rates significantly (*p*<0.01) improving by 12.88 and 30.44%, respectively. Our findings demonstrate that WTE, especially at medium and high concentrations, significantly (*p<*0.01) enhances oxidative stress resistance versus the control group. Furthermore, females showed greater tolerance to H₂O₂-induced stress than males (Figure 4), suggesting a sex-dependent difference in stress resilience among fruit flies (51).

### 3.5 Role of WTE in modulating dietary intake and enhancing athletic capacity in fruit flies

**Figure 5:**
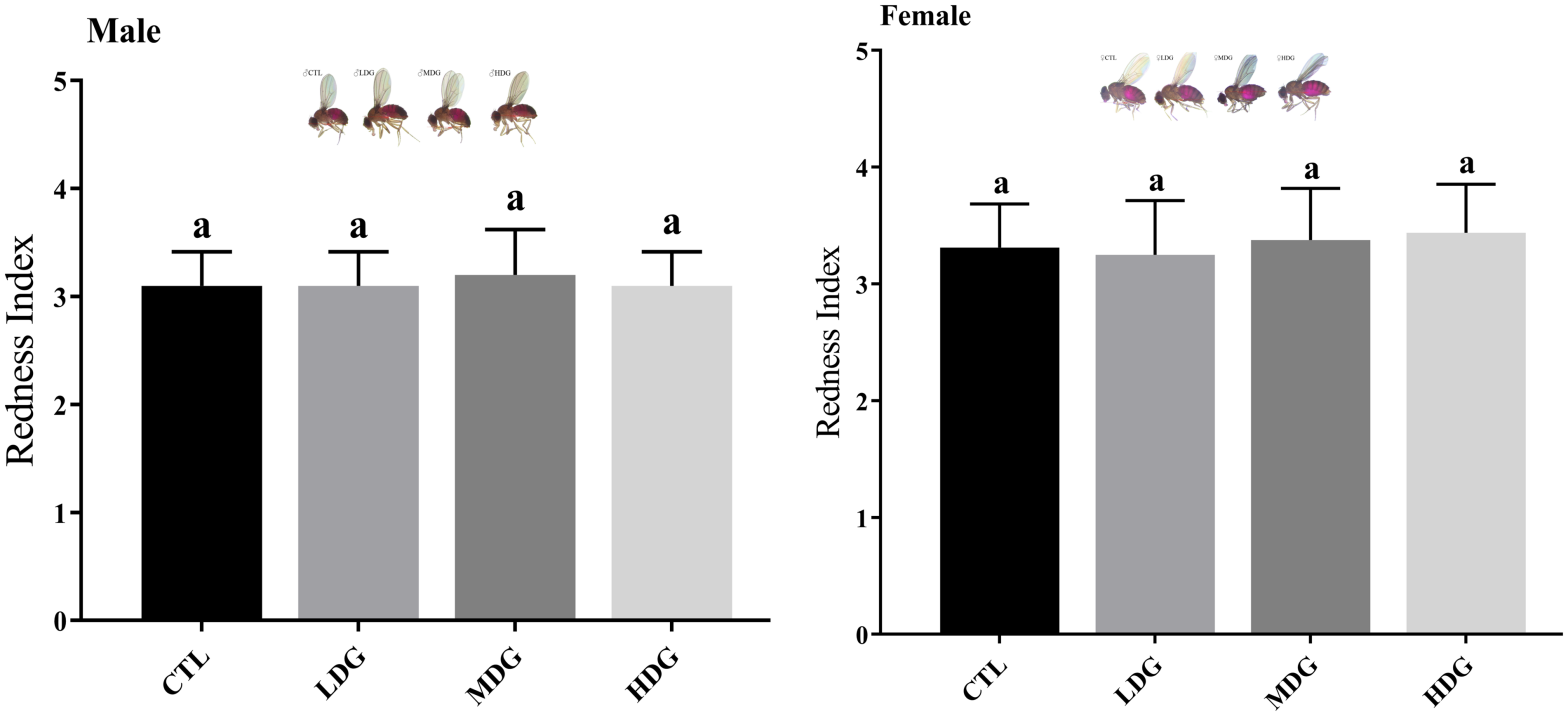
Abdominal redness index of *Drosophila melanogaster* fed diets supplemented with white tea extract (WTE) at concentrations of 0.5 (LDG), 1.0 (MDG), and 3.0 mg/mL (HDG). Data are presented as mean ± SD (*n* = 3). Different lowercase letters indicate statistically significant differences among groups (*p* < 0.05). CTL, Control group; LDG, MDG, and HDG: low-, medium-, and high-dose WTE groups, respectively.

Red coloration index of the abdomen, assessed by counting the number of visibly red abdominal segments, serves as an indicator of feeding behavior in fruit flies. A consistent level of redness suggests comparable food consumption among the groups (52). After WTE supplementation, no changes were observed in the feeding behavior of the flies; all groups exhibited three red abdominal segments, suggesting that the anti-aging effects were not related to altered dietary behavior (Figure 5). Statistical analysis showed no significant difference (*p*>0.05) in terms of stomach erythema grading between WTE-treated groups and the control, showing comparable food consumption across sexes and treatments. Dietary restriction has been reported to considerably enhance lifespan in flies according to previous studies (53). Our feeding assay results ruled out dietary restriction as a factor contributing to lifespan extension, supporting that the increased longevity was due to WTE supplementation rather than reduced food intake. Additionally, flies did not avoid the WTE-containing medium, confirming that their feeding patterns remained unchanged.

**Figure 6:**
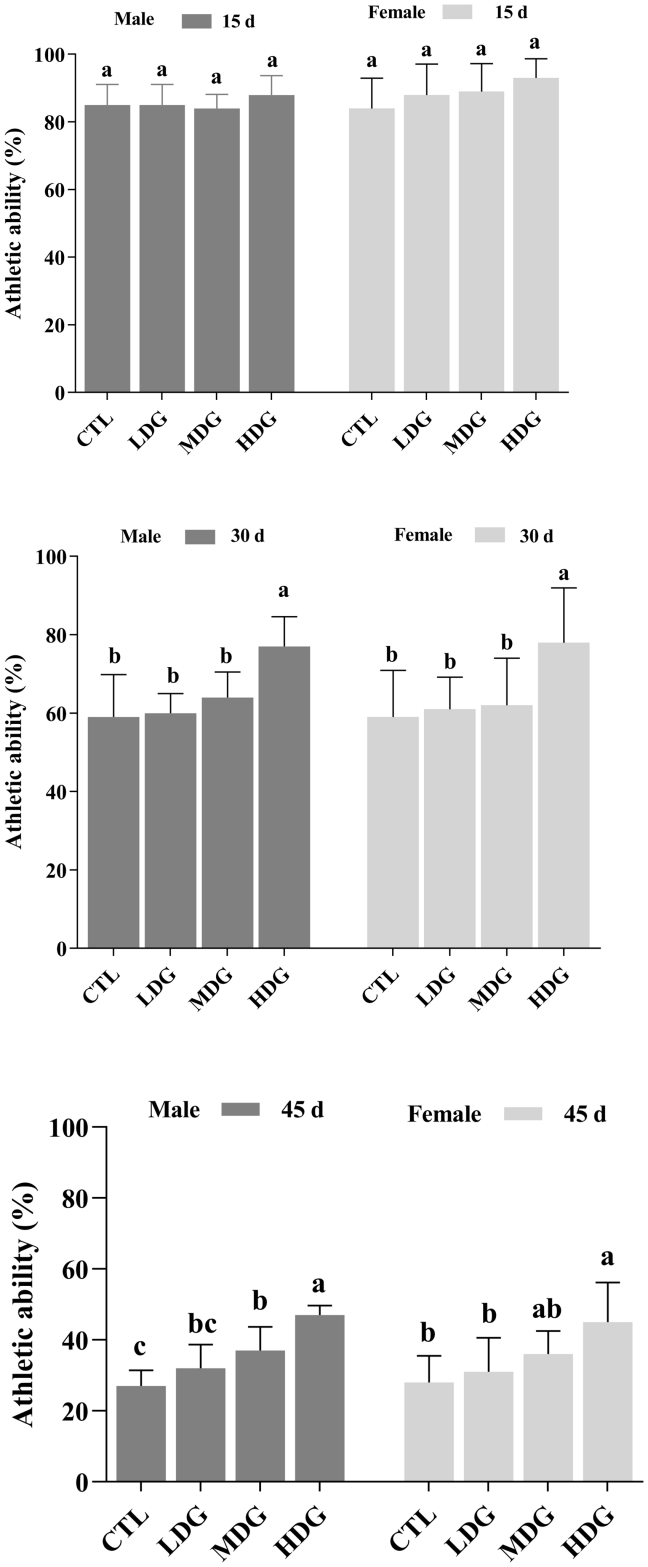
Effect of different concentrations of white tea extract (WTE) on the climbing performance of male and female *Drosophila melanogaster* at specified time points. Flies were fed diets supplemented with 0.5 (LDG), 1.0 (MDG), and 3.0 mg/mL (HDG) WTE. Data are presented as mean ± SD (*n* = 5). Different lowercase letters indicate significant differences among groups on the same day (*p* < 0.05). CTL, Control group; LDG, MDG, and HDG: low-, medium-, and high-dose WTE groups, respectively.

Metabolic slowdown and reduced physical activity are widely observed during aging in animals as well as humans. In fruit flies, athletic ability, particularly vertical climbing, decreases significantly with age and serves as an important indicator of organismal deterioration (54). By days 30 and 45, fruit flies in the CTL showed a significant (*p*<0.01) reduction in athletic performance (Figure 6). However, dietary supplementation with WTE significantly (*p*<0.01) improved the locomotor abilities of both male and female fruit flies, with the most pronounced effects observed in the HDG. On day 30, athletic performance increased by 30.51% in males and 32.20% in females within the HDG. Remarkably, by day 45, these improvements reached 74.07 and 60.71%, respectively (*p*<0.01). Additionally, flies receiving WTE supplementation exhibited more agile movements compared to other groups, indicating that WTE enhances exercise capacity in both sexes.

### 3.6 Role of WTE in modulating antioxidant enzymes in fruit flies

**Figure 7:**
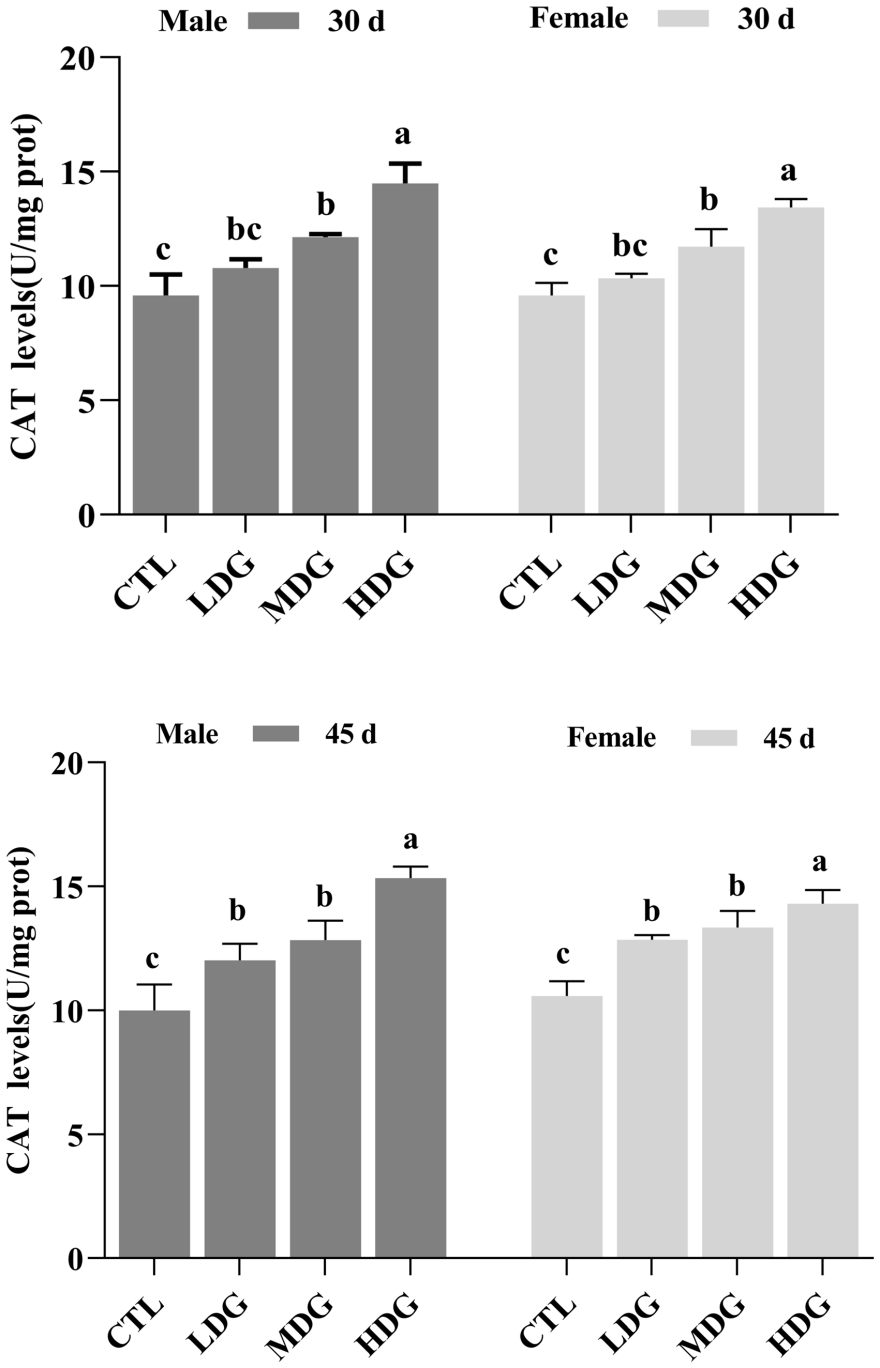
Catalase (CAT) activity in *Drosophila melanogaster* supplemented with white tea extract (WTE) at 0.5 (LDG), 1.0 (MDG), and 3.0 mg/mL (HDG) on Days 30 and 45. Data are presented as mean ± SD (*n* = 3). Different lowercase letters on the same day indicate statistically significant differences among groups (*p* < 0.05). CTL, Control group.

Reactive oxygen species (ROS) are common free radicals produced during normal metabolic activities. Under oxidative stress induced by external factors, excessive ROS can overwhelm the organism’s antioxidant defenses, accelerating aging and mortality (55). The ROS-detoxifying enzyme system plays a vital role in mitigating oxidative damage by eliminating surplus free radicals, thus maintaining redox balance and slowing aging. This study evaluated the effects of different doses of WTE supplementation on catalase (CAT) and total superoxide dismutase (T-SOD) activities in fruit flies. CAT levels increased with age in both male and female flies. On day 30, the LDG showed no significant change (*p*>0.05) in CAT activity compared to controls, whereas MDG and HDG exhibited significant (*p*<0.01) increases in both sexes (Figure 7). By day 45, CAT activity rose proportionally with WTE amount. When treated with 3 mg/mL, male CAT activity significantly (*p*<0.01) increased by 53.40%, reaching 15.3 U/mg protein from a baseline of 10.0 U/mg protein and 35.16% in females (from 10.6 to 14.3 U/mg protein).

**Figure 8:**
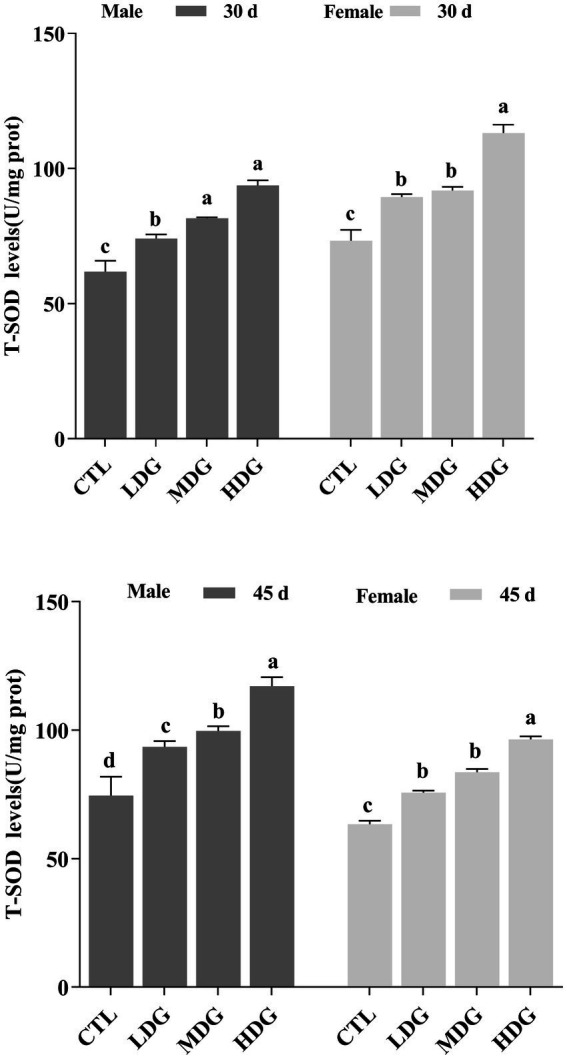
Total superoxide dismutase (T-SOD) activity in *Drosophila melanogaster* after dietary supplementation with white tea extract (WTE) at 0.5 (LDG), 1.0 (MDG), and 3.0 mg/mL (HDG) on Days 30 and 45. Data are presented as mean ± SD (*n* = 3). Different lowercase letters on the same day indicate statistically significant differences among groups (*p* < 0.05). CTL, Control group.

Dietary supplementation with WTE markedly, dose-dependent increase in T-SOD activity in fruit flies, with a more pronounced effect observed in females (Figure 8). On day 45, flies receiving 3 mg/mL WTE showed significant elevations in T-SOD activity by 52.08% (*p*<0.05) in males and 57.14% (*p*<0.01) in females. These results suggest that WTE enhances the activity of key antioxidant enzymes, confirming its potent antioxidant effect in fruit flies. SOD and CAT are vital endogenous antioxidants that form the core of the body’s defense system against free radicals, thereby contributing to the delay of aging. This study found that both CAT and T-SOD activities increased with age in a dose-dependent manner across all groups, with female fruit flies showing significantly (*p*<0.01) higher T-SOD activity than males. Previous research has indicated that extracts of green and black tea can similarly enhance antioxidant potential in fruit flies (56). Flavonoids, comparable to tea antioxidants such as catechins and theaflavins, exert significant antioxidant functions (57). Consistent with these findings, our results demonstrate that WTE boosts Levels of enzymes involved in oxidative stress defense and partially reduces MDA synthesis, which may contribute to lifespan extension in fruit flies.

### 3.7 Role of WTE in modulating levels of oxidative by-products

**Figure 9:**
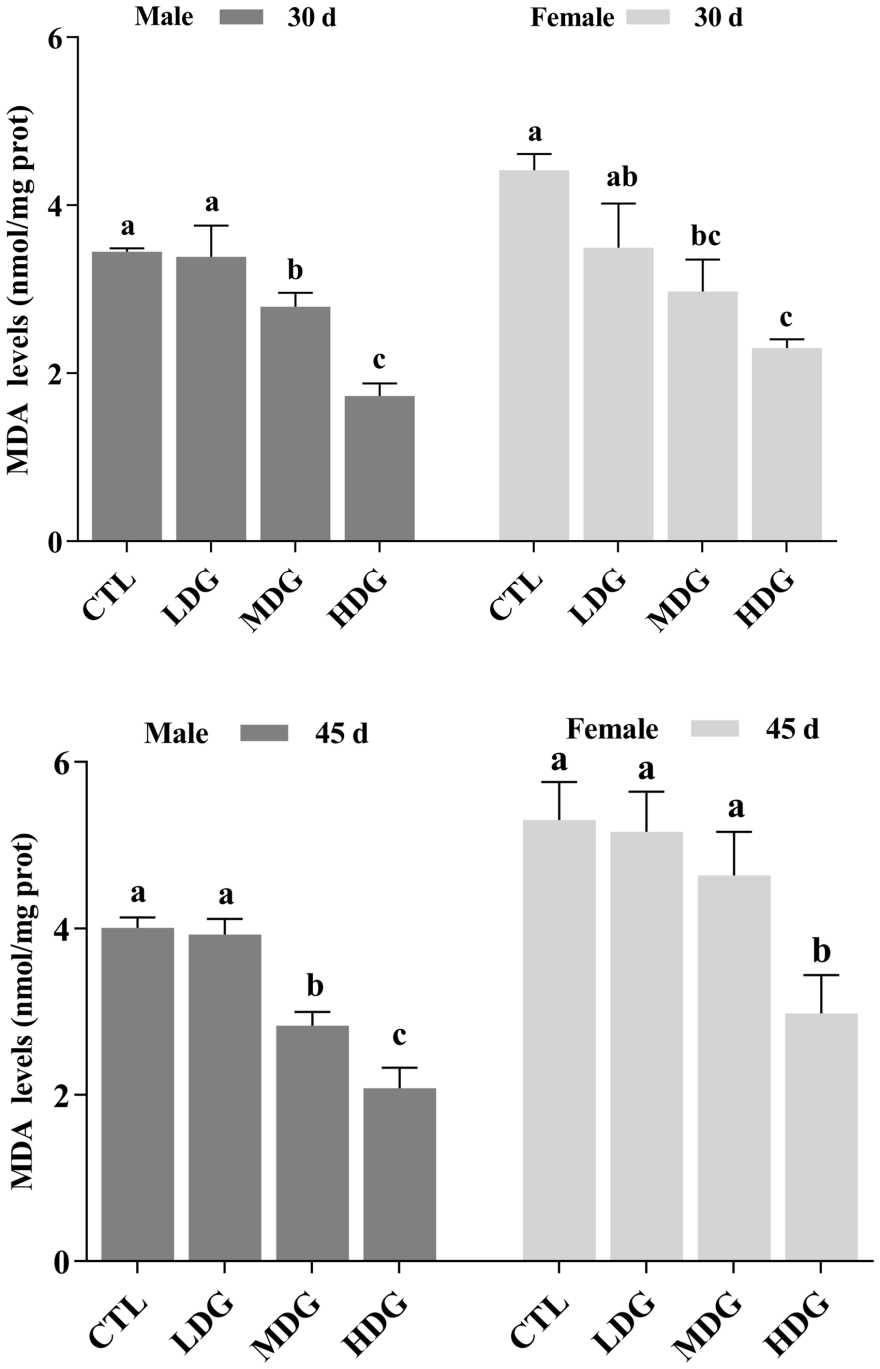
Effect of varying concentrations of white tea extract (WTE) on malondialdehyde (MDA) content in *Drosophila melanogaster* measured on Days 30 and 45. Data are presented as mean ± SD (*n* = 3). Different lowercase letters on the same day indicate statistically significant differences among groups (*p* < 0.05). CTL, Control group; LDG, MDG, and HDG, Diets supplemented with 0.5, 1.0, and 3.0 mg/mL WTE, respectively.

MDA, a byproduct of lipid peroxidation caused by free radicals, can promote cross-linking and polymerization of macromolecules such as proteins and nucleic acids, leading to cellular membrane damage and cytotoxic effects (58). In this study, MDA levels in both male and female fruit flies from the MDG and HDG were significantly (*p*<0.05) decreased on day 30 (Figure 9). By day 45, fruit flies in the HDG showed a further significant (*p*<0.01) reduction in MDA levels compared to controls, reflecting a decline in antioxidant defense during natural aging. Flies in the LDG exhibited a slight decrease in MDA (1.72% in males and 1.98% in females), but this was not statistically significant (*p*>0.05). At 3 mg/mL WTE, MDA levels dropped significantly (*p*<0.01) by 49.86% in males and 47.96% in females. These findings indicate that WTE effectively reduces lipid peroxidation and oxidative harm in fruit flies. This concurs with earlier reports showing that goji berry polysaccharides effectively lengthen fruit fly lifespan and decrease MDA content (59).

### 3.8 Role of WTE in regulating relative expression

**Figure 10:**
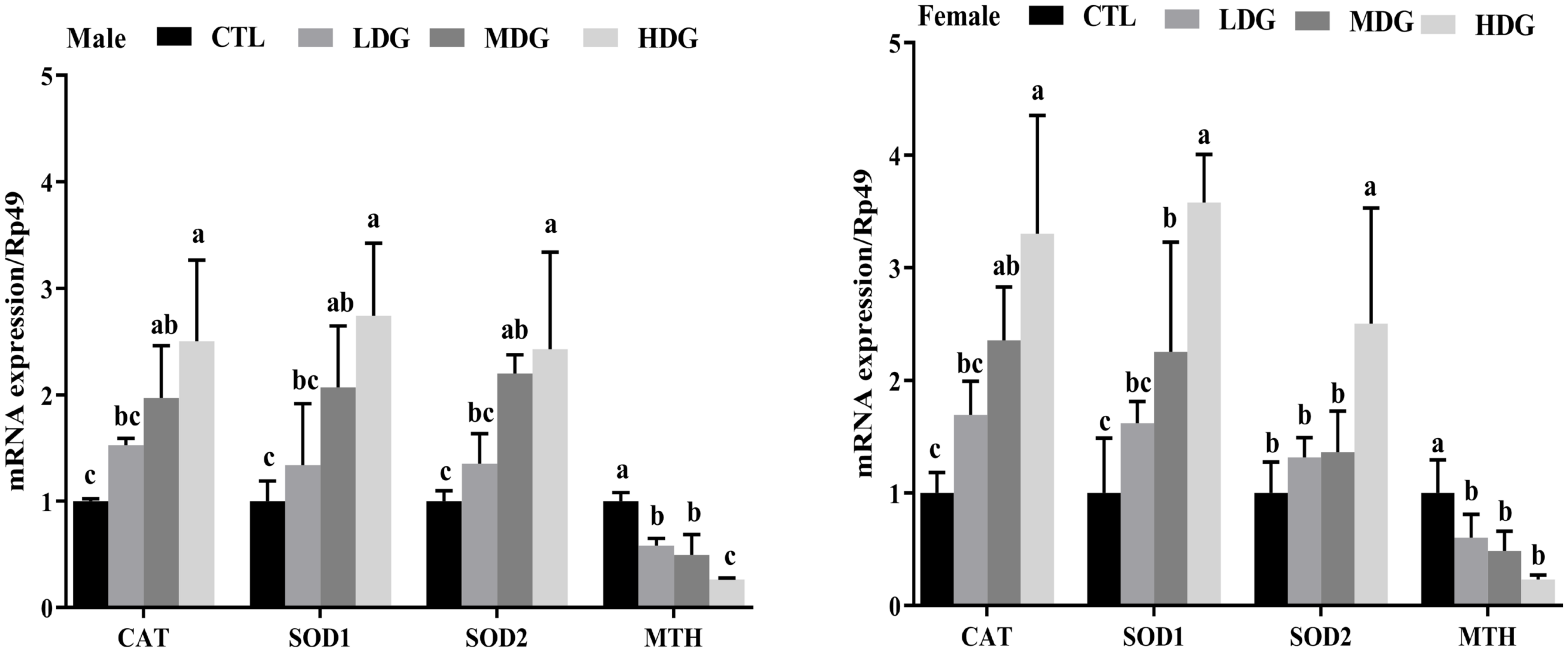
Expression levels of antioxidant-related genes (CAT, SOD1, SOD2, and MTH) in *Drosophila melanogaster* fed diets supplemented with white tea extract (WTE) at 0.5 (LDG), 1.0 (MDG), and 3.0 mg/mL (HDG). Data are presented as mean ± SD (*n* = 3). Different lowercase letters above bars indicate statistically significant differences among groups (*p* < 0.05). CTL, Control group.

Quantitative PCR was performed to assess the impact of WTE on the expression of antioxidant-related genes (CAT, SOD1, SOD2, and MTH) in fruit flies (Figure 10). Results showed that CAT and SOD1 were significantly (*p*<0.01) upregulated in both MDG and HDG across male and female flies compared to the control. SOD2 expression was significantly (*p*<0.01) increased in male fruit flies within the MDG and HDG. Notably, the expression of the MTH gene, which typically increases with age and contributes to aging by diminishing antioxidant defenses, was significantly (*p*<0.01) decreased in all WTE-treated male and female groups compared to the controls. The significant reduction in MTH mRNA levels across the dose groups corresponded with the observed lifespan extension in fruit flies.

A positive association was observed between the antioxidant activities of fruit flies and the transcriptional levels of antioxidant-related genes, including CAT, SOD1, and SOD2. The increase in CAT and T-SOD enzyme levels induced by WTE suggests that its antioxidant properties contribute to lifespan extension. WTE improves the antioxidant enzyme system in fruit flies by upregulating these genes, which enhances the organism’s ability to neutralize oxidative stress and delay aging. This proposed anti-aging mechanism aligns with findings by Cai et al. (29), who demonstrated similar functional properties of peptides derived from crimson snapper scales. Similarly, Kang et al. (44) reported that blueberry extract’s age-delaying effects involve modulation of antioxidant gene expression.

## 4 Conclusion

WTE is a bioactive, health-beneficial ingredient, mainly due to its abundance in flavonoid content, including rutin, myricetin, quercetin, and kaempferol. It demonstrated strong *in vitro* antioxidant activities against DPPH, hydroxyl radicals, superoxide anions, and ABTS radicals. The study showed that supplementation with WTE improved the physical functions of fruit flies and extended their lifespan. This lifespan extension was closely linked to an improved antioxidant defense system, evidenced by decreased MDA levels, elevated activities of T-SOD and CAT enzymes, and increased expression of antioxidant genes (CAT, SOD1, and SOD2). These findings highlight WTE’s potential as a functional dietary antioxidant and anti-aging agent, indicating promising applications in the food and health sectors. Given that the WTE comprises a diverse range of bioactive compounds, the isolation, purification, and structural elucidation of the flavonoid constituents responsible for its anti-aging properties are imperative. This meticulous process can facilitate the identification of the key bioactive molecules and elucidate their intricate structure–activity relationships. Future research will validate the anti-aging potential of white tea extract using mammalian models like mice to provide more robust biological evidence. These experiments will determine if the beneficial effects observed in fruit flies can be extrapolated to higher organisms and clarify the underlying molecular and physiological mechanisms.

## Data Availability

The raw data supporting the conclusions of this article will be made available by the authors, without undue reservation.
